# Effectiveness of Iloprost in the Treatment of Bone Marrow Edema

**DOI:** 10.7759/cureus.10547

**Published:** 2020-09-20

**Authors:** Hacı Bayram Tosun, Abuzer Uludağ, Sukru Demir, Sancar Serbest, Mehmet Mete Yasar, Kadir Öznam

**Affiliations:** 1 Orthopaedics, Medipol University, Istanbul, TUR; 2 Orthopaedics, Adiyaman University Faculty of Medicine, Adiyaman, TUR; 3 Orthopaedics and Traumatology, Fırat University, Elazıg, TUR; 4 Orthopaedics and Traumatology, Kırıkkale University Faculty of Medicine, Kirikkale, TUR; 5 Orthopaedics and Traumatology, Adıyaman University Faculty of Medicine, Adıyaman, TUR; 6 Orthopaedics and Traumatology, Medipol University School of Medicine, Istanbul, TUR

**Keywords:** bone marrow edema, iloprost, treatment

## Abstract

Background and objective

Bone marrow edema (BME) is a rare condition caused by insufficient osseous blood supply and may result in severe pain that has adverse effects on patients’ life. To date, various conservative treatments have been recommended for the treatment of BME, including analgesics, immobilization of the affected extremity, and iloprost infusion. The aim of this retrospective study was to investigate the effectiveness of parenteral iloprost therapy in the treatment of BME detected in different skeletal locations.

Materials and methods

This retrospective study included 23 patients (17 men and six women) with BME who were classified as stage I-III according to the Association Research Circulation Osseous (ARCO) classification. BME was localized to the proximal femur in 13 (56.5%), the distal femur in four (17.4%), tarsal bone in four (17.4%), and tibial plateau in two (8.7%) patients. The mean age of the patients was 46.7 years and all the patients were evaluated with the Visual Analog scale (VAS), Functional Mobility Scale (FMS), and MRI.

Results

A significant improvement was observed in the post-treatment VAS and FMS scores of all patients compared to their pre-treatment scores. Moreover, the edema regressed completely in 60.9% of the patients at three months of MRI control. No serious side effects were observed during the treatment in any of the patients. However, transient side effects including headache, arrhythmia, and flushing were observed in five patients.

Conclusion

The present study indicated that iloprost therapy is an effective and safe option in the treatment of BME patients, particularly in the reduction of severe pain that has adverse effects on patients’ social life, regardless of ARCO staging. Moreover, this therapy could be particularly useful in reducing pain, improving functional recovery, and achieving complete regression of the edema on MRI in ARCO stage I-II patients.

## Introduction

Bone marrow edema syndrome (BMES) is an uncommon and self-limited syndrome characterized by extremity pain. BMES can either occur in isolation or be accompanied by avascular necrosis (AVN) [1]. BMES is characterized by an increase of interstitial fluid within the bone, and unless corrected, it can lead to necrosis with the local collapse of the bone [2,3]. The syndrome mostly affects middle-aged men and young women and often occurs in the bones of the hip, knee, ankle, and foot. The symptoms usually appear at rest or during physical activity and worsen progressively [1-3].

The primary goal of the treatment of BMES is to reduce pain and disease duration. Treatment options are limited, including both conservative and surgical techniques [1]. Although numerous conservative techniques including pharmacological agents such as warfarin, enoxaparin, statins, bisphosphonates, and acetylsalicylic acid, and other treatment modalities including extracorporeal shock wave therapy (ESWT), hyperbaric oxygen therapy (HBOT), electro-stimulation, and pulsed electromagnetic field therapy (PEMFT) have been performed in the treatment of BMES, none of them has been shown to prevent the disease effectively and delay femoral head collapse, and some of them have been shown to have multiple side effects [4-8].

Iloprost is a pharmacological agent that has been recently shown to reduce bone marrow edema (BME) by achieving significant improvement in the symptoms [9,10]. The prostacyclin derivative iloprost is a vasoactive substance used in the treatment of pulmonary hypertension, vasculitis, and vascular occlusion [11]. Iloprost leads to arterial and venous vasodilation and also inhibits the activity of red blood cells (RBC), leukocytes, and platelets. It inhibits edema formation by limiting the hydrostatic pressure in the terminal vascular bed and also prevents the recurrence of edema by improving the flow characteristics of the blood and by regulating the endothelial function [9,12]. Additionally, iloprost inhibits platelet aggregation and attenuates the formation of reactive oxygen species (ROS) and leukotrienes in the vascular bed [9,13]. Iloprost is known to have no growth-stimulating effect in mature osteoblasts [14], and its effect on edema formation associated with bone necrosis remains unclear [9,10].

The aim of this retrospective study was to investigate the effectiveness of parenteral iloprost therapy in the treatment of BMES detected in different skeletal locations.

## Materials and methods

The retrospective study included 23 patients who underwent parenteral iloprost therapy due to BMES detected in different skeletal locations between 2018 and 2019. The study was approved by the local ethics committee and was performed in accordance with the ethical standards laid down in the Declaration of Helsinki. All patients were informed about the treatment and written informed consent was obtained from each patient. The study included patients who were detected with BME in different skeletal locations on MRI regardless of the coexistence of AVN and had severe pain that resulted in adverse effects on their daily life activities.

BME is radiographically classified according to the Association Research Circulation Osseous (ARCO) classification [7]. Both plain radiographs and MRI scans [T1-/T2-/short inversion time inversion recovery (STIR)-weighted] were obtained both before and at three months after iloprost therapy [15]. ARCO allows grading from a reversible BME (ARCO I) to irreversible local necrosis (ARCO II) with subchondral fractures (ARCO III) and secondary osteoarthritis (ARCO IV) [16,17]. Moreover, the resolution of BME after treatment was evaluated based on MRI images (stage 0: normal stage; stage 1: edema in one-third; stage 2: edema in two-thirds; stage 3: edema in all; and stage 4: AVN in addition to stage 3) [3].

All patients were administered an intravenous infusion of 20 μg/ml of iloprost (Ilomedin®, Bayer Schering, Berlin, Germany) in 500 ml of sodium chloride solution, given over a period six hours, on five consecutive days [3-6,9,10]. All patients were admitted to the hospital and monitored closely for possible adverse effects. Adverse effects were categorized as severe (hypotension, arrhythmia, bleeding, thromboembolism, acute respiratory distress syndrome, pulmonary edema, allergic reactions with systemic clinical signs, shock), and minor (flush, erythema, headache, nausea, phlebitis) [9,18]. In addition to iloprost treatment, all patients received 70 mg of alendronate per week, 150 mg of acetylsalicylic acid once a day, and cholecalciferol with calcium carbonate once a day for three months.

All patients with ARCO stage IV edema, cardiac arrhythmia, atrial fibrillation or pulmonary hypertension, blood coagulation disorders, chronic infectious diseases, pregnancy, tumor diseases, gastrointestinal ulcer, heart failure, recent myocardial infarction (MI) or unstable angina pectoris, anticoagulant therapy such as warfarin or heparin, or those who were under the age of 18 years were excluded from this study [5,9,18]. Also, patients with an ARCO IV stage and those receiving ozone therapy, core decompression, and hyperbaric oxygen therapy were excluded from the study.

Medical history and clinical examination were documented both before iloprost treatment and at six weeks, three months, and at the latest follow-up after the initiation of the therapy. The pain level was documented by using a Visual Analog Scale (VAS) ranging from 0-10 points with 0 points representing no pain and 10 points representing the worst imaginable pain. Moreover, the Functional Mobility Scale (FMS) was used for functional assessment of the patients (score 0: full activity; score 1: walking with assistance; score 2: walking with assistance for short periods; score 3: walking with assistance for activities of daily living/appointments only; score 4: confined to a wheelchair; and score 5: bedridden).

Weight-bearing was allowed as far as tolerable, and those with hip problems were advised to use crutches. All patients were advised to stay away from contact sports activities and heavy work for three months. The patients were followed up for six months and were then contacted by telephone to obtain information.

Statistical analysis

Statistical analyses were performed using IBM SPSS Statistics software version 16.0 (IBM, Armonk, NY). A two-tailed p-value of <0.05 at a confidence interval (CI) of 95% was considered significant for all the analyses. The normal distribution of continuous variables was analyzed using the Shapiro-Wilk test. Continuous variables were compared using the Wilcoxon signed-rank test as they were all normally distributed, and the VAS scores were analyzed using the Friedman test.

## Results

The study included 17 men and six women with a mean age of 46.7 years. BME was localized to the right side in 43.47%, to the left side in 30.43%, and was localized bilaterally in 26.1% of the patients. The mean duration of symptoms was 3.34 months (range: 1-9 months). In patients with bilateral involvement, BME was localized to the proximal femur in five and to the tarsal bone in two patients. BME was idiopathic in 74% of the patients; 4.3% of the patients were pregnant, and 21.7% of them had a history of corticosteroid use (Table 1). The ARCO stages of the patients ranged from stage I to stage III (Table 2).

**Table 1:** Baseline characteristics of study participants SD: standard deviation

Characteristics	Value (n=23)
Age in years; mean ±SD (range)	46.7 ±9.3 (22-64)
Sex (female/male)	6/17
Risk factors	
Corticosteroid use	5
Pregnancy	1
Idiopathic extremity; proximal femur; distal femur; tibia plateau; tarsal	17; 13; 4; 2; 4

**Table 2:** Association Research Circulation Osseous (ARCO) stage

ARCO stage	Characteristics	Number of patients
I	Reversible bone marrow edema	8
II	Irreversible local necrosis	6
III	Irreversible local necrosis with subchondral fractures	9
IV	Secondary osteoarthritis	Excluded from study

The mean pre-treatment VAS score was 9.08 ±0.9 and the mean post-treatment VAS score at the last follow-up was 2.43 ±2.35 (p<0.001). The VAS scores in patients with ARCO stages I, II, and III improved significantly at three and six months after the treatment compared to pre-treatment scores (p<0.05). There were no significant differences in the VAS scores in all ARCO stages between three and six months (p<0.05). Also, although a remarkable reduction was observed in the VAS scores of ARCO stage I patients in 10 days after treatment, no significant difference was found (p>0.05) (Table 3).

A significant improvement was observed in the FMS scores of all patients compared to their pre-treatment scores. The mean pre-treatment FMS score was 0.26 ±0.44 and the mean post-treatment FMS score was 2.43 ±0.5 (p<0.001).

**Figure 1:**
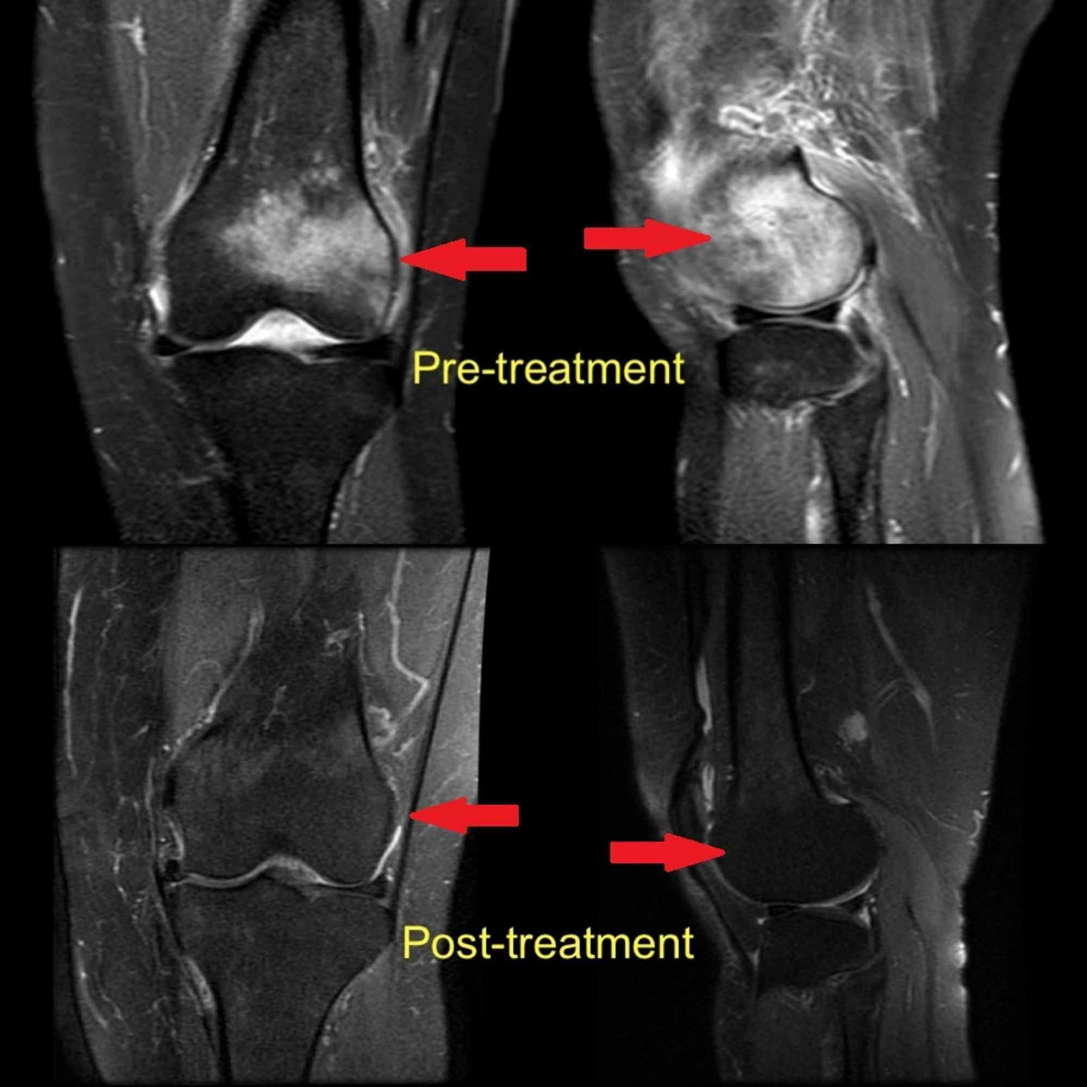
MRI appearance of bone marrow edema of the lateral femoral condyle (arrows) MRI: magnetic resonance imaging

**Figure 2:**
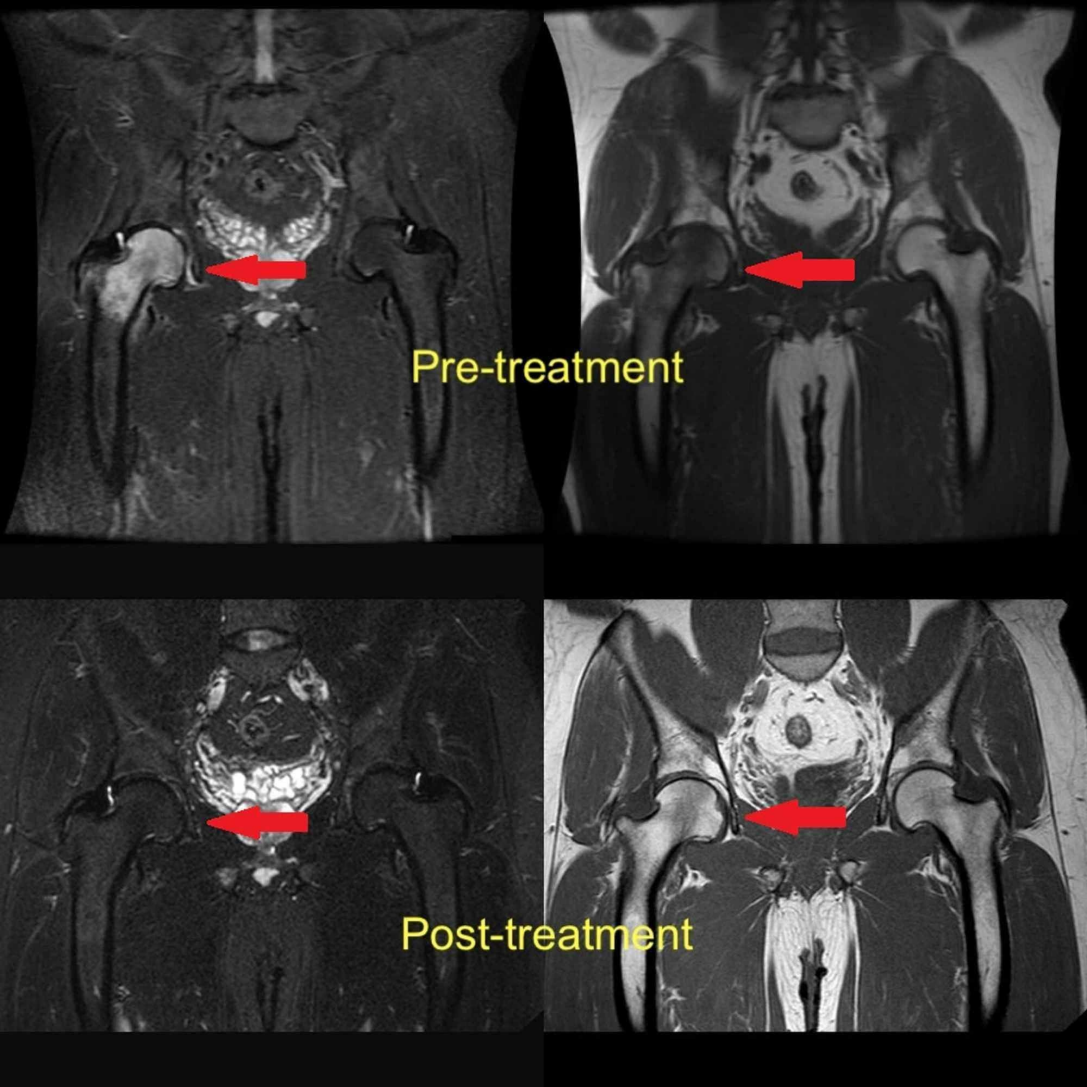
MRI appearance of bone marrow edema of the proximal femur (arrows) MRI: magnetic resonance imaging

At the three-month follow-up, MRI revealed a significant reduction of edema in all patients; while the edema regressed completely in 14 (60.9%), a minimal BME was observed in four (17.4%), and moderate BME was observed in five (21.7%) patients. In the last follow-up visit at six months, MRI showed a slight decrease in AVN findings in four patients without BME and showed signs of AVN progression such as demarcation, the ‘double-line sign’, or bone collapse in five patients with ARCO II-III stage, and these patients had the same complaints (Figures 1, 2).

No serious side effects were observed during the treatment in any of the patients. However, transient side effects including headache, arrhythmia, and flushing were observed in five patients.

## Discussion

Insufficient osseous blood supply can result in BME and/or AVN. The prostacyclin analog iloprost is a treatment option used for improving bone blood perfusion [3-9,10]. Jäger et al. administered iloprost therapy in 95 patients and reported that the pain levels decreased and the functional scores improved in all the patients. The authors also noted that although healing was not possible in advanced stages of AVN, iloprost therapy can still contribute to pain relief and improve joint function [18]. Disch et al. used iloprost for the treatment of 16 patients with isolated BME and 17 patients with BME associated with necrosis of the proximal femur and reported that both groups showed a significant and lasting improvement in objective and subjective clinical criteria. The patients in both groups also showed remarkable improvement in the range of movement, pain, and the extent of the area of edema, irrespective of the baseline findings. During the five-day period of hospitalization, 83% of the patients showed an improvement in symptoms, regardless of the presence of necrosis, and no significant difference was found between the two groups. The authors proposed that the primary aim of iloprost therapy should be to prevent further spread of necrosis by reducing edema formation [3]. In our study, the patients presented with BMES in different skeletal locations and were administered parenteral iloprost therapy; the patients also had severe pain that gradually worsened and had adverse effects on their daily life activities beginning from day 10 of the onset of the illness. Of note, the VAS scores assessed at months three and six after therapy and the FMS scores assessed at the last follow-up showed significant improvement compared to baseline values. Moreover, BME disappeared completely in 60.9% of the patients at three months of MRI control.

Meizer et al. treated 104 patients with BME in different skeletal locations with iloprost therapy and reported that patients with idiopathic (ischemic) and traumatic BME showed significant improvement in pain at rest and pain under stress as well as in MRI findings at four months after therapy [19]. Another study by Meizer et al. reported that on MRI, 20 patients showed complete normalization, four showed no change, and three showed worsening of the MRI pattern [13]. Claßen et al. [20] and Röhner et al. [6] have suggested that iloprost therapy is a safe and effective method in the treatment of AVN, particularly in patients with an early ARCO stage (stages I and II). Hörterer et al. treated 42 ARCO stage I-III patients with BME of the foot and ankle and reported that the treatment resulted in a 60% pain and 80% edema reduction after three months [21]; however, the patients indicated having residual impairment after two years. Aigner et al. administered iloprost therapy in six patients with BME of the talus and reported that the edema disappeared completely on MRI and also a significant and rapid improvement was observed in functional scores at three months after therapy [9]. Zippelius et al. [22] used iloprost for the treatment of 15 ARCO stage I-II patients with BME of the knee joint and reported that 80% of the patients showed complete regression of the edema at three months of MRI control and three patients underwent additional surgery in later periods [3].

Another study by Zippelius et al. evaluated 19 patients that underwent iloprost therapy due to BME of the hip joint and reported that the patients showed significant improvement in the Western Ontario and McMaster Universities Osteoarthritis Index (WOMAC) and VAS scores during long-term follow-up, and 79% of the patients showed complete regression of the edema at three months of MRI control [5]. At 6-12 months' follow-up, four ARCO stage II patients progressed to ARCO stage III and IV; among them, two patients required total hip replacement despite core decompression and iloprost therapy. Based on these findings, the authors suggested that iloprost therapy could be a viable option in the treatment of BME patients in ARCO stages I-II [5]. In our study, although all the patients showed significant improvements in the VAS and FMS scores, 21.7% of the patients (particularly those with ARCO stages II and III) showed progression on MRI.

Various treatment modalities have been recommended for the treatment of BME, including analgesics, immobilization of the affected extremity, bisphosphonates, and systemic intravenous iloprost complemented with vitamin D and calcium supplementation [5,6-8]. In a previous systematic review, Roth et al. indicated that although iloprost is highly effective in reducing BME and the accompanying pain in ARCO stage I-II patients, it may not be appropriate for the treatment of subchondral fractures [7]. The authors also noted that the bisphosphonate (alendronate) therapy delayed the damage and collapse of the femoral head and also reduced the pain by inhibiting bone resorption. Based on these findings, the authors suggested that iloprost and alendronate could be used for the conservative treatment of BME and that other pharmacological and physical therapies were not appropriate for the treatment of BME [7]. In our study, alendronate, cholecalciferol with calcium carbonate, and acetylsalicylic acid therapies were administered as a complement to iloprost therapy. Given that our findings indicated that the treatment led to a rapid improvement in the symptoms in patients who had severe pain that had an adverse effect on their daily life activities, we believe that these additional therapies made a remarkable contribution to the treatment. Since distinguishing self-limited BME from the BME that occurs at the onset of AVN is highly difficult, this progress could be aided by eliminating the risk factors, initiating support therapies, and limiting weight-bearing on the affected extremity in the early stage.

Our study has some limitations. It consisted of a small number of patients, only included iloprost therapy, had a retrospective design, and had no control and/or comparative group. More prospective, randomized, controlled studies are needed to further investigate and explore an effective treatment method for BME patients.

## Conclusions

In conclusion, the present study indicated that iloprost therapy is an effective and safe option in the treatment of BME patients, particularly in the reduction of severe pain that has adverse effects on patients’ social life, regardless of ARCO staging. Moreover, this therapy could be particularly useful in reducing pain, improving functional recovery, and achieving complete regression of the edema on MRI in ARCO stage I-II patients.
